# Corneal blindness in Plato’s cave: the acting forces to prevent and revert corneal opacity. Part I: epidemiology and new physiopathological concepts

**DOI:** 10.5935/0004-2749.20200102

**Published:** 2024-02-11

**Authors:** Amanda Pires Barbosa, Monica Alves, João Marcello Fortes Furtado, Leidiane Adriano, Luis Fernando Nominato, Lara Cristina Dias, Marina Zilio Fantucci, Adriana de Andrade Batista Murashima, Eduardo Melani Rocha

**Affiliations:** 1 Research Core in Ocular Physiopathology and Therapeutics and Department of Ophthalmology, Otorhinolaryngology and Head & Neck Surgery, Faculdade de Medicina de Ribeirão Preto, Universidade de São Paulo, Ribeirão Preto, SP, Brazil; 2 Department of Ophthalmology and Otorhinolaryngology, Faculdade de Ciências Médicas, Universidade Estadual de Campinas, Campinas, SP, Brazil

**Keywords:** Blindness/epidemiology, Blindness/prevent & control, Blindness/therapy, Corneal opacity, Cegueira/epidemiologia, Cegueira/prevenção & con trole, Cegueira/terapia, Opacidade da córnea

## Abstract

The burden of corneal blindness and visual deficiency can be felt worldwide. Its association with several endemic diseases such as childhood blindness, trauma, infectious keratitis (including variants caused by herpes, hanseniasis, and fungi), vitamin A deficiency, diabetes mellitus, and other dry eye syndromes reflects its poorly understood underlying mechanisms and suggests that the actual frequency of the disease is underestimated. The low effectiveness of preventive and therapeutic strategies against corneal scarring or deformity predicts a high frequency of patients with corneal blindness in the future. Corneal blindness is associated with environmental factors and socioeconomic limitations that restrain health assistance and maintain a modest efficiency of the current therapeutic strategies for resolving corneal diseases in large-scale programs. We present here a critical review of the concepts associated with corneal blindness that need to be considered when planning strategies to prevent and treat corneal blindness worldwide (to be able to leave Plato’s cave, where corneal blindness is encaged.

## Abstract (pt)

O problema da deficiência visual e da cegueira corneal abrange o mundo todo e corresponde à quarta causa de cegueira e deficiência visual, com acometimento estimado de mais de 16 milhões de pessoas. A associação com várias doenças endêmicas, como cegueira infantil, trauma, ceratites infecciosas (incluindo herpes, hanseníase e fungos), hipovitaminose A, diabetes mellitus e outras causas de síndromes de olho seco, indicam que a verdadeira frequência é subestimada e que os diferentes mecanismos são pouco conhecidos. A baixa eficácia na prevenção e tratamento da cicatriz e deformidade da córnea permite antecipar que a prevalência da cegueira corneal irá crescer no futuro. As razões para o aumento da cegueira corneal envolvem fatores ambientais, limitações socioeconômicas para ampliar a assistência à saúde e a modesta eficiência das estratégias terapêuticas para resolver o problema em grande escala. O presente trabalho traz uma revisão crítica dos conceitos associados à cegueira corneal. Essa análise é uma etapa necessária para preparar o caminho com o objetivo de deixar a caverna que encarcera a cegueira corneal, em analogia ao mito de Platão, e melhorar as estratégias para prevenir e tratar a cegueira corneal em escala mundial.

## INTRODUCTION

After a rapid overview of the medical literature and lectures presented in clinical conferences about corneal diseases, opacity, and corneal blindness, one may arrive at four conclusions: a) corneal blindness is a rare and distant problem; b) the causes are predictable, and the events leading up to corneal blindness are preventable; c) most of the causes of corneal injury are treatable, and the blindness outcome is avoidable; d) therapeutic approaches are very effective and long lasting^(1-3)^. These optimistic conclusions persist because most of the publications addressing corneal diseases fail to mention combined frequencies of the causes of corneal blindness, the rate of success and long-term outcomes of the available treatments, and the limitations to such treatments in the less technologically advanced and most populated regions of the planet. In the following sections, we will argue that corneal blindness is not well defined, that the above conclusions are wrong, and that a reduction of the burd en of corneal blindness will not be achieved in large segments of the population using the present strategies.

The analogy with Plato’s cave in this work is justified by the apparent scenario where knowledge about corneal blindness is “fixed,” which brings to mind the fictional condition described by Plato in approximately 380 B.C. in “The Republic.” In a dialogue between Socrates and his brother Glauco, Socrates described individuals captive in a cave from a very young age, whose only sources of information are noises and shadows projected onto the cave wall in front of them. Prevented from leaving or even looking back, they are unable to understand their situation, until one of them escapes and makes contact with the outside world for the first time. After returning to the cave, the fugitive reports his experience to his former cave-mates and offers to help them escape. However, the captive individuals are skeptical and refuse the opportunity to be free^(4)^.

The journey to understand the causes, frequency, mec hanisms, and treatments of corneal opacity and blindness is reminiscent of the allegory of Plato’s cave in several ways. In the corneal blindness cave, the four assumptions enunciated above (a-d) are fed and supported by information produced by “normal” science, as defined by Thomas S. Kuhn in his work addressing the structure of scientific revolutions^(5)^. In the cave, corneal opacity is a minor issue, well addressed in terms of public health and therapeutic strategies, and most symptoms can be solved with the refinement of certain therapeutic and surgical strategies. However, as in Plato’s cave, new knowledge is opening opportunities to address the challenge of corneal blindness, and this new information, which redefines the limits of our understanding, is met with skepticism.

Our aims with this review are to show data on the prevalence and mechanisms of corneal blindness, to explain why the problem is not improving, and to highlight the frustrating limitations associated with current treatment modalities. In the final section, we will show the perspectives for future corneal blindness treatments. In continuing with the analogy of Plato’s cave, the concepts brought forth by researchers, who left the “normal” science on corneal blindness, have been received with doses of skepticism.

### The burden of corneal blindness

The World Health Organization (WHO) recently red efined visual impairment as a visual acuity >0.5 (or 20/40) and blindness as >0.05 (or 20/400) in the better-seeing eye, using the concept of “presented” instead of “best-corrected” visual acuity. This classification does not distinguish treatable from untreatable blindness or functional low vision^(6,7)^.

**Figure 1:**
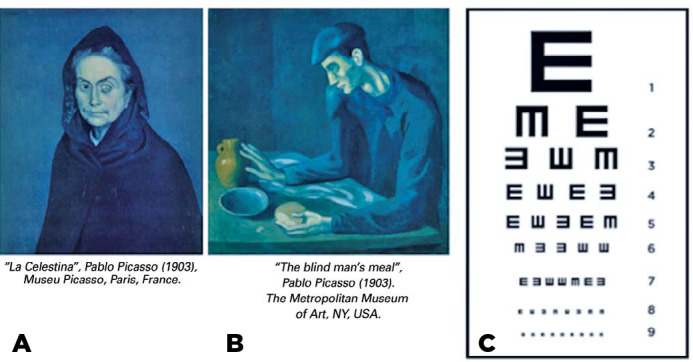
Pablo Picasso’s portraits from his blue phase, showing (A) a woman with an opaque left cornea and a normal right eye and, therefore, not matching the criteria for blindness (”Celestina”) and (B) a man without eye globes, using his hands to identify the food in front of him and who is thus blind not just by Picasso’s but also by the World Health Organization’s definition (“The Blind Man’s Meal”). (C) Visual acuity chart used to determine visual impairment and blindness with vision in the better eye and in the worse eye lower than <0.5 and <0.05, respectively.

We can illustrate the difference between a blind eye and a blind person by referring to two portraits painted by Pablo Picasso in 1903. “Celestina” has an opaque (left) cornea but a normal right eye. In “The Blind Man’s Meal,” the character is using his hands to identify the food, and the face lacks the globe of the eye. These paintings illustrate the distinction between a blind person (“The Blind Man’s Meal”) according to the WHO definition and a person with a blind eye (Figure 1).

**Figure 2:**
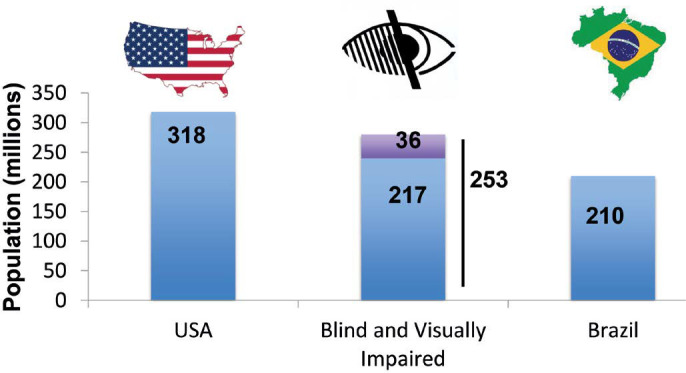
Comparison of the population of USA and Brazil with the estimated number of individuals with blindness (top of the middle bar) and visual impairment (bottom of the middle bar) throughout the world.

From recent estimates, the number of blind individuals in the world is approximately 36 million, and the number of those with moderate to severe visual deficiencies is 217 million^(8)^. Taken together, this is a population comparable in size to those of the largest countries such as Brazil (211 million people) and the USA (327 million people)^(8-10)^ (Figure 2).

The most frequent causes of visual impairment and blindness are uncorrected refractive errors and cataracts. Retinal diseases (including diabetic retinopathy), corneal blindness (summing trauma, infection, childhood blindness, vitamin A deficiency, and trachoma), and glaucoma present similar numbers of affected individuals^(9,11)^. Glaucoma and retinal diseases have been addressed with technological improvements allowing early diagnosis and options for treatment that has reduced their prevalence among the causes of blindness during the past few decades^(12-16)^. Data collection about blindness prevalence in population studies is oriented towards easily treatable causes in situations where two or more conditions contribute similarly to the blindness or visual impairment diagnosis^(17)^.

Cataracts and refractive errors persist as large causes of visual impairment and blindness in studies and projections due to barriers against accessing eye health facilities and making technologies available in areas distant from big cities^(18-21)^. Models of rapid interventions that leave communities without an established service have clearly failed to prevent or reduce visual impairment because of cataracts and refractive errors^(22)^.

Grouped causes of corneal blindness and visual impairment may amount to a total of 16 million affected people, but the real numbers are difficult to obtain because of differences in search methods, regions evaluated, grouping, and analyses, as well as the uncertainty intervals of the estimated rates^(9,11)^. A study in the Amazon region of Brazil revealed that pterygium, combined with corneal opacity, accounted for 12% of cases of blindness. Another study in Latin America showed corneal opacities as responsible for 4% of the cases of blindness, in contrast to the results of another study in São Paulo, Brazil, where corneal opacity and pterygium were not identified as causes of blindness^(7,23-27)^.

Infectious keratitis (caused by bacteria, fungi, viruses, or parasites) can cause corneal opacities and blindness. However, the frequency of bilateral cases that would fit the definition of visual impairment and blindness has not been comprehensively registered, and the global prevalence remains unknown. Studies have suggested that infectious keratitis is the thirdor fourth-leading cause of corneal blindness, behind pterygium, trauma, and surgery^(28)^. Other frequent causes of corneal blindness identified in referral clinics and tertiary hospitals such as keratoconus and dysgenetic and dystrophic diseases (Peters’ anomaly, sclerocornea, and endothelial dystrophies) are underrepresented in population studies because they may be included in different groups as corneal opacities, refractive errors (keratoconus in the astigmatic group), and childhood blindness, and a systematic criteria to allow merging data from different epidemiological studies is lacking. Beyond this issue, an under-registered number of individuals with monocular visual impairment or binocular asymmetric corneal disease also exist. These observations indicate that more individuals will progress to corneal blindness in the future, and the numbers will also grow due to better registration techniques^(8,26)^. The incidence of trachoma has shown a considerable decline in recent years, credited to the SAFE (surgery for trichiasis, antibiotics against Chlamydia, facial cleaning, and environmental improvements) strategy^(14,29)^. In addition, other risk factors for corneal blindness and visual impairment tend to grow in the future because of factors, including increased life spans, limited access to treatments, and underestimated causes related to dry eye disease (DED): pterygium, pollutants, excessive ultraviolet light exposure, and the presence of high amounts of toxic agents in the environment^(7,9,14,18,26-31)^.

**Figure 3:**
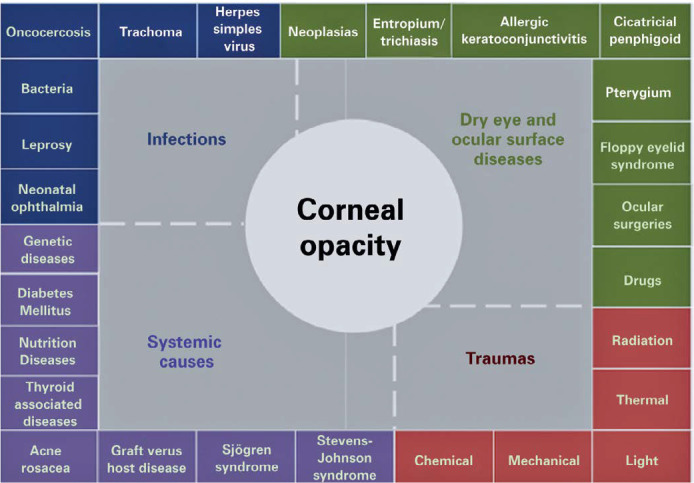
Panel with potential causes of corneal diseases that cause blindness, grouped according to similar mechanisms of action.

DED is a frequent and increasingly prevalent condition in the population^(32)^ that is an underestimated risk factor for corneal blindness and is frequently associated with worse outcomes in diseases resulting in corneal blindness^(33,34)^. Corneal opacities and consequent blindness may occur in diseases that cause DED (Figure 3). Vitamin A deficiency and trachoma are among the conditions that, when combined, lead to DED and corneal blindness^(26,35)^. The prevalence of DED ranges between 5% and 50%, depending on diagnostic and inclusion criteria and geographic regions^(32)^. The comorbidity and pathologic correlations between DED and corneal blindness deserve clarification. Moreover, DED causes, as potential risk factors for corneal blindness, need further studies.

### Knowing the causes and managing the consequences

Could the rates of corneal blindness due to opacity be reduced if we knew its risk factors and demographics? Intuition and epidemiology say yes; that is what is observed in the epidemiologic triangle elaborated in the XIX century and its modern variations, which summarize the quote: “to know, to prevent”^(36,37)^. Studies on the demographics of an individual with eye trauma over three decades have found practical difficulties: Patients that attend the clinic for eye trauma seem to be all young men with similar risk factors. In other words, observations from the 1980s to the 2000s have revealed that young, male handworkers are more likely to suffer eye traumas (frequently wounding the cornea), mostly in the workplace, leading to similar functional, social, and economic consequences in all of them^(38)^. In that report, approximately 80% of patients were not using protective devices, and 30% had had at least another ocular trauma. These data are similar in other regions throughout the world^(16,28,39,40)^. The same observations can be extrapolated to infectious keratitis, especially that caused by filamentary fungi, a devastating corneal infection that occurs mostly in young, male agricultural workers, and which presents few therapeutic options and carries poor prognoses for the cornea and vision^(41-45)^. Ibrahim et al. found that fungal keratitis has a seasonal prevalence associated with low humidity and low temperatures; approximately 38% of the cases resulted in therapeutic corneal transplants (15% with eye globe evisceration), and approximately 60% have a blind eye by the first year of follow-up^(42,44)^. Less common but also strongly associated with a specific risk factor is Acanthamoeba keratitis, where the association is with bad compliance with proper contact lens care^(46-48)^.

These observations indicate that preventive measures can be used to reduce the occurrence of ocular trauma and infectious keratitis, two major causes of corneal blindness^(26,28)^ (Table 1). However, the frequency of these illnesses and the same patient profiles can be found throughout the world. Therefore, the myth of Plato’s cave applies to corneal blindness, revealing the shadows on the wall to be topics on the size of the problem and its “accidental” causes. Two of the previous assumptions can be refuted by data: corneal blindness is not rare, and despite knowledge on its epidemiology, the incidence remains high.

**Table 1:** Individual or collective strategies to prevent corneal blindness

Type of prevention	Description	Effects	Author, year
Individual	Seatbelt use and air bags in automobile transportation	Reducing the severity and the grade of visual loss by ocular trauma in car accidents	Rao SK et al, 2008^(49)^
	Standard specifications for eye protective devices at work and during sportive activities	Significant reduction in eye injuries, face, and head attachments due to impact or penetration of a paintball	Tseng VL, et al, 2014^(50)^
	Ocular prophylaxis with 1 % silver nitrate, 0.5% erythromycin ointment, or 1% tetracycline hydrochloride to all newborns	Reduction of gonorrheal ophthalmia neonatorum incidence	Paediatr Child Health, 2002^(51)^
Collective	Legislation for the control of fireworks use	Reduction in the frequency of eye injuries in states in the USA with a “Model Law” banning all fireworks, except those used in public displays	Kuhn F, 2010^(52)^
	Vitamin A distribution, breastfeeding promotion, food fortification, and counseling regarding dietary changes	Reduction of keratomalacia incidence	Oliva MS et al, 2012^(53)^
	Ivermectin widespread distribution	Reduction in the incidence of onchocerciasis in endemic areas	Kim YE et al, 2015^(54)^
	SAFE plan	Reduction in the frequency of trachoma and parallel rates of corneal blindness due to trachoma	Travers A et al, 2013^(55)^

SAFE= Surgery, Antibiotic, Face washing, and Education

### Established and useful concepts on corneal structure and physiology

The cornea is a transparent organ that fills 1 cm^2^ of the area in front of the eyeball. It has a hemispherical format and less than 1 mm in thickness. It is almost 90% transparent and mostly composed of water and acts as a shield for the eye globe. Given its fragile profile, a major challenge is to understand how the cornea resists and responds to environmental and external aggressions^(56)^.

The surface is protected by a tear film with a complex and variable composition^(57)^. Its ingredients are produced in the exocrine glands present in the ocular surface and the goblet and epithelial cells in the epithelial layer. The tear film flows as a result of the eyelids blinking at an average pace of 10 to 20 times per minute, which renews the tear film volume of 10 µL at a rate of 1 µL/ minute^(57)^. This mechanism allows for nutrition, protection, and stimuli to the cells of the eye that are replaced throughout the life of an individual^(57,58)^.

The five layers of the cornea have well-described roles that allow it to act as a barrier for the whole eye and as an efficient optical lens. The corneal epithelium prevents microorganism and toxic agent invasions, the endothelium controls the water content, and the stroma gives transparency and dioptric power to match the needs of the eye. The stroma is found between two membranes: Bowman’s membrane, on the external side of the cornea, separates the stroma from the epithelial-layer basement membrane, and Descemet’s membrane, on the internal side of the cornea, separates the stroma from the endothelium^(59)^. Improvements and growth in the number of lamellar corneal transplants have drawn the attention of cornea surgeons allowing for the characterization of a pre-Descemet membrane, also called Dua’s membrane^(60)^, a more compact corneal layer, with few keratocytes found between Descemet’s membrane and the posterior part of the stroma. After some skepticism, this sixth layer is being gradually accepted, and it has been found to be associated with the mechanism of corneal hydrops and the elastic resistance of the descemetocele, at the same time being used as a safety variable during surgical techniques for deep anterior lamellar keratoplasty (a form of corneal transplant)^(61,62)^.

The transparency of the cornea is supported mainly by its avascularity. Blood and lymphatic vessels grow in the cornea from the corneal limbus in response to aggressions and inflammation^(63)^. The avascularity is sustained by the permanent expression of soluble vascular endothelial growth factor receptor (sVEGFR) in the ocular surface and in the stroma^(64)^. When sVEGFR is suppressed, new vessels grow in the cornea^(64)^.

A dense network of nerve fibers detects external, harmful stimuli and modulates the reactions of the cor nea^(65-67)^. These nerve fibers are linked to the environment by a family of transient receptor potential (TRP) channels activated by environmental variations in temperature, pH, osmolarity, and mechanical stimuli^(68)^. The responses escalate based on the intensity of the stimulus and trigger signals capable of attracting inflammatory mediators that activate wound healing processes^(69,70)^. Interestingly, aggressions limited to the epithelium are relatively benign, and the body is able to restore the epithelial homeostasis a short time after the initial offense; however, aggressions that hit the stroma leave long lasting or even permanent scars in the stromal layer^(71)^. Two recently described mechanisms help to explain this response: The first one involves activation of the transient receptor potential vanilloid 1 (TRPV1) in epithelial cell cultures by osmolarity, temperature, and chemical challenges, which induce secretion of inflammatory cytokines (IL-6 and IL-8) through the Mitogen-Activated Protein Kinase signaling pathway, but also induces corneal epithelial cell migration through epidermal growth factor receptor transactivation^(72-75)^. In addition, the activation of TRPV channels in keratocytes present in a deeper corneal layer (the stroma) promotes the secretion of transforming growth factor-beta and induces the production of collagen, which is responsible for stromal scar formation^(70,76-79)^. Taken together, these findings indicate that superficial damage to the epithelium prompts fast wound healing and the preservation of transparency despite a painful and inflammatory process and deeper injuries to the corneal stroma, which destroys the nerve network and jeopardizes the eye globe integrity or triggers a mechanism of new vessel growth, and strengthens the globe wall (corneal stroma). The delicate structure that provides transparency is abdicated in favor of a scar, which is an opaque and stronger barrier against external injuries. To attend that natural rule of corneal transparency, mechanisms are in place to actively and wisely protect the cornea against neovascularization, where dense innervation not only provides high sensitivity but also inhibits neovascularization; however, the opposite occurs in response to corneal damage, denervation, or nerve network damage that allows for neovascularization, which, in turn, inhibits reinnervation^(80)^.

Connections among the corneal layers also respond to persistent injuries. One example is the chronic use of contact lenses, which leads to changes in the shape of endothelial cells; the other example is bullous keratopathy^(81,82)^, which induces stromal edema due to a lack of deturgescence control resulting from the loss of endothelial cells and from inflammatory events in the ocular surface that induce neovascularization and loss of limbal stem and goblet cells^(81)^. In this disease, the repercussions to the stroma and ocular surface may be explained by endothelial cell responses to a hypotonic environment that induce a paracrine secretion of inflammatory cytokines, mediated also by the TRPV1 channels^(78,83,84)^.

Taken together, this information helps to clarify the initial mediators and steps in the mechanisms underlying the fast superficial lesion repairs without inflammation. On the other hand, the lesions that hit the stroma or the endothelium, either from the external or internal side of the eye and whose effects last for a long period of time, can induce extensive inflammatory processes and a permanent corneal scar.

Epithelial replacement is crucial for faster wound healing, and corneal epithelial stem cells asymmetrically distributed in niches on the limbal region, called the palisades of Vogt, prevent the development of lesions to the stromal layer^(85-87)^. During the last three decades, explanations have been provided for how these stem cells renew and replace corneal basal epithelial cells^(58,88)^. What is not clear is the manner in which epithelial wound healing occurs independently of the corneal limbal epithelial cells in certain animal eye lesion models and clinical conditions^(89-93)^.

In summary, the five recent observations about the corneal structure and the response to injuries are examples of relevant information brought to the Plato’s cave of corneal blindness that, once overcoming the initial wave of skepticism and being applied to treatments for corneal blindness, will change the epidemiological scenario described above (Table 2). Interestingly, a review article authored by Tseng and Tsubota in 1997 advanced some of these concepts, although without the steps or molecular details^(58)^.

**Table 2:** Corneal structure updates and transparency mechanisms outside of Plato’s cave - antigo Box 1

Topic Observation		Authors
1 Avascularity Active mechanism of corneal vascularity inhibition		Ambati et al, 2006, Ferrari e t al, 2013^(66,82)^
2 Sensitivity Ion channels with distinct sensorial and repair responses in different corneal layers		Zhang et al, 2007 and Okada et al 2013^(71,75)^
3 Structure A dua membrane protects the deep cornea		Dua et al, 2013^(61)^
4 Inflammation Interchangeable effect of damage to the inner face of the cornea on the ocular surface		Uchino et al, 2006^(82)^
5 Epithelial stem cells Mechanisms of corneal epithelial replacement based on the limbal niche of stem cells		Tseng & Tsubota, 1997, Dua et al, 2005^(59^, ^87)^

### History and present limitations of using penetrating keratoplasty for the treatment of corneal opacity

The major strategy for fixing an opaque, perforated, or melted cornea is a replacement of the organ. Conceptualizations and improvements in the technique have been described in other reviews with minimum variations in terms of historical details^(94-96)^. The first physician credited with mentioning the possibility of corneal replacement was Galen, in Greece, sometime between 130 and 200 A.D. In the XVIII century, different authors conceptualized the possibility of curing corneal blindness. Among them were Erasmus Darwin and Guillaume Pellier de Quengsy^(94-96)^. During the XIX century, a heterologous strategy (i.e., using the cornea from other animal species) was evaluated by several authors, and in the XX century, the first homologous transplant took place. The remarkable advances in the understanding of the biology of grafts, from the 1960 Nobel Prize awarded to Peter Medawar to the studies on mechanisms of immunotolerance, clarified the players involved in the success and failure of corneal transplantations^(63,97)^. The penetrating keratoplasty technique improved considerably after the 1960s with the introduction of four key elements: eye banking, surgical microscopy, 10-0 nylon sutures, and post-operatory corticotherapy^(94,98)^.

The limitations that impede the success of this procedure can be summarized by two points (Table 3):

**Table 3:** Limitations for keratoplasty to revert corneal opacity as a health plan strategy - antiga Box 2

Limitations	Analysis	Authors
1 Limited availability of corneal donation for transplants	Estimated 180,000 keratoplasties/year for an estimated total of 16 million people with corneal blindness	Pascolini & Mariotti 2012, Gian et al., 2016^(9,100)^
2 Limited survival of corneal transplants compared to the patients’ life expectancies	The corneal transplant survival is around 12 years, and the average life expectancy for patients is longer	Tan et al, 2008, and Kontis et al, 2017^(102,105)^

1) The limited availability of corneas for all cases of corneal blindness. An average of 180,000 corneal transplants is performed every year worldwide, far less compared with the 16 million patients with corneal opacities and low vision or blindness^(9,11,99,100)^. In fact, the estimated number of donor corneas or penetrating keratoplasties available every year covers only 1 out of every 70 cases awaiting this treatment^(99)^. A considerable effort to increase the number of donor tissues and the number of facilities to treat those people would be necessary to revert corneal blindness with this strategy. Considering these numbers, the capacity must grow several times over to be able to meet the present demand.

2) The survivor curve of corneal transplants worsens the limitation of donor corneas. Studies have revealed that under favorable conditions, the half-life of a graft is approximately 12 years; however, in adverse situations such as massive inflammation (therapeutic) or perforated cornea (tectonic), the mean survival time of the grafts is as low as 5 and 2 years, respectively^(101-103)^.

Insisting on the strategy of corneal transplants under these unfavorable conditions is an unwise option to revert corneal blindness^(100,104-108)^. Since the 1970s, clinical scientists and researchers have worked together to develop alternatives or complementary strategies to corneal transplants^(95,106)^. The most recent options, including pharmaceutical and surgical alternatives to avoid corneal blindness, will be addressed in a subsequent review, but lying beyond the shadows and the skepticism are useful and innovative strategies for treating corneal blindness, as supported by the concepts presented above.

This review summarized the problem of corneal blindness, addressing epidemiological flaws and the mechanisms of the major causes of this disease with the limitations of relying on corneal transplants to provide a cure and reduce the number of blindness conditions worldwide. We believe our review sheds a light on the shadows inside the cave and summarizes the work of researchers who, upon leaving the cave and observing beyond its opening, have returned with rich pieces of information for understanding the size of the problem, its detailed physiopathology, and the fragility of the present therapeutic strategies for treating corneal blindness.
